# When Teachers Mentalize: A Mixed-Methods Analysis of Teacher Responses to Disruptive Classroom Behaviour

**DOI:** 10.3390/children13070911

**Published:** 2026-07-09

**Authors:** Gali Chelouche-Dwek, Aitana Miracle, Brenda McHugh, Neil Dawson, Matthew Hillman, Peter Fonagy

**Affiliations:** 1 Research Department of Clinical, Educational and Health Psychology, University College London, London WC1E 6BT, UK; 2 Anna Freud Centre, London N1 9JH, UK

**Keywords:** mentalization, mentalization-based interventions, teacher practices, alternative provision, disruptive classroom behaviour

## Abstract

**Highlights:**

**What are the main findings?**

- Teachers’ use of Mentalization, Down-regulation, and Playing with reality techniques was associated with a significantly higher likelihood of resolving disruptive classroom incidents; each additional MBI applied was associated with a robust additive increase in resolution odds, stable across all severity levels.
- Only one pairing—Mentalization combined with Playing with reality—showed a tentative, exploratory synergistic association beyond their individual contributions, suggesting a possible role for playfulness as a vehicle for reflective work in the classroom.

**What are the implications of the main findings?**

- Teacher-centred MBIs were associated with the resolution of disruptive behaviour in this exploratory observational study, generating hypotheses for the future design of evidence-based teacher training programmes grounded in mentalization theory.
- Translating MBT principles into educational contexts requires careful attention to theoretical fidelity; Validation, Collaboration, and Scaffolding held potential but require re-operationalisation before wider implementation.

**Abstract:**

**Background**: Disruptive classroom behaviour remains a persistent challenge for teachers, peers, and students alike. Mentalization-based interventions (MBIs) have shown promise in addressing such behaviour, yet research has predominantly focused on student-centred or school-wide approaches, leaving teacher-centred applications underexplored. **Aims**: This study investigated (1) whether teachers’ use of MBIs during incidents of disruptive behaviour was associated with incident resolution, and whether this varied by severity, and (2) how MBIs were enacted in naturalistic classroom settings. **Methods**: Interactions between students and teachers were observed across two classrooms in a London-based alternative provision school, yielding 259 documented incidents of disruptive behaviour and 815 coded teacher–student interaction sequences—the primary unit of analysis. Teacher responses were coded for six predefined MBIs (including Mentalization; shifting focus to underlying mental states, and Playing with reality; encouraging flexible perspectives through humour or playfulness), using a validated scheme with high inter-rater agreement across all coding dimensions (Cohen’s κ > 0.75). Incident outcomes (resolved vs. unresolved) served as the dependent variable, with severity as a moderator. Thematic analysis of classroom dialogue characterised how MBIs were enacted in practice. **Results**: Several MBIs were associated with a significantly higher likelihood of incident resolution, while others were less effective. Only one intervention pairing—the simultaneous use of Mentalization and Playing with reality—showed a tentative synergistic association which should be treated as exploratory. Robust additive effects were observed across incident severity. Qualitative analyses highlighted how MBIs were delivered, including differences in tone, style, and strategy. **Conclusions**: In this naturalistic observational study, teachers’ use of MBIs was associated with the resolution of disruptive behaviour in classrooms. These findings extend understanding of how MBT principles can be translated into educational contexts and provide preliminary evidence to inform future research on teacher-centred MBIs, which will require controlled designs before programme development.

## 1. Introduction

Disruptive behaviour in the classroom refers to any actions by students that interfere with the teaching and learning process, ranging from relatively minor behaviours—such as inattention or off-task activity—to more severe forms, including defiance, oppositionality, and verbal or physical aggression [1,2,3].

Disruptive behaviour is the leading cause of both suspensions and permanent exclusions in the UK, which are associated with a range of negative outcomes, including lower academic achievement, unemployment, and involvement in serious violence [4,5,6]. These behaviours also pose significant challenges for educators, with teachers frequently reporting feeling underprepared to manage disruption [7,8], and difficulty doing so a well-documented source of burnout and attrition from the profession [9,10,11].

Given the far-reaching consequences for students, teachers, and schools alike, there is an urgent need for effective, evidence-based approaches to help teachers manage disruptive behaviour in the classroom. A promising approach that has emerged in clinical settings is Mentalization-Based Treatment (MBT) [12], which shifts attention from external behaviour to the internal mental states that underpin it.

### 1.1. Mentalization-Based Treatment

MBT is a form of psychotherapy that integrates principles from attachment theory, psychodynamic psychotherapy, and cognitive-behavioural therapy [12]. Originally developed by Bateman and Fonagy [13,14] for the treatment of borderline personality disorder (BPD), MBT is grounded in the theory that individuals with BPD struggle with emotional regulation and interpersonal functioning due to vulnerability to breakdowns in mentalization—the capacity to interpret and understand one’s own and others’ mental states, such as beliefs, intentions, desires, and emotions [12]. MBT seeks to strengthen this capacity, particularly within attachment contexts, to help individuals recognise that behaviour—both their own and others’—is driven by underlying mental states, by fostering a safe, supportive therapeutic relationship—a secure base from which individuals can explore their own and others’ mental states [15].

Although initially developed for adults with BPD, MBT has since been adapted for a range of clinical populations, including children, adolescents, and families [16], as well as for various psychological disorders [17]. Of particular relevance to education, a growing body of evidence suggests that MBT may be effective in addressing disruptive behaviour in children and adolescents [18,19,20,21]. For example, Hauschild et al. [20] found preliminary support for the feasibility of MBT with adolescents with conduct disorder, with post-treatment improvements in diagnoses and empathy, though the small sample size limits conclusions about efficacy. More robust evidence comes from Halfon et al. [19], who conducted a randomised controlled trial in 222 school-age children and found that mentalization-based treatment for children (MBT-C) produced significant reductions in externalising problems—including rule-breaking and aggression—compared to an active control condition.

The consistency of these findings across populations provides a strong rationale for exploring how MBT principles might be adapted for non-clinical settings, such as schools. MBT-C is especially relevant to the present study. Beyond adapting the approach for younger populations, it is distinctive in working not only with the child but with the child’s wider support system, including parents, school, and the broader social network, to build a mentalizing environment around the child [22]. Applying a clinical framework in a school is therefore not a wholesale transfer of therapy into the classroom, but a translation of its core mentalizing stance into the brief, real-time exchanges that make up ordinary teaching.

### 1.2. Mentalization-Based Interventions in Schools

Given the central role schools play in children’s socio-emotional development, they offer a cost-effective and wide-reaching platform for psychological interventions [23]. While MBT is a structured, long-term psychotherapy developed for clinical settings, mentalization-based interventions (MBIs) represent shorter, more flexible adaptations of mentalization theory suited to non-clinical environments such as schools [24,25]. MBT refers to the manualised clinical treatment, whereas MBIs are the broader set of interventions that apply mentalizing principles outside formal therapy, including in schools. MBIs share common ground with trauma-informed and reflective-teaching approaches but are defined here by their explicit focus on the child’s mental states.

A growing body of evidence suggests that school-based MBIs are associated with medium to large improvements across a range of psychological and behavioural outcomes, including reductions in disruptive behaviour [24]. These interventions have been implemented at multiple levels, including student-centred MBIs, which aim to strengthen children’s mentalizing capacities and socio-emotional skills, and school-wide programmes, which seek to embed mentalizing principles across the broader school climate. A third, largely understudied level is teacher-centred MBIs, which target teachers’ capacity to interpret and respond to student behaviour through a mentalizing lens. Such interventions may offer a valuable middle ground: student-centred approaches can be limited by their narrow focus on individual skill development, whereas school-wide programmes often face challenges of feasibility and scalability.

The only formally evaluated teacher-centred MBI programme to date is the Thoughts in Mind (TiM) project [26], which involved two teachers and their classes (46 school-aged children). One teacher received training in the implementation of mentalizing strategies in everyday classroom practice—consisting of two three-hour sessions at the start of the school year and two supervisions across the year—while the other participated in a matched control activity. Students in the TiM training group demonstrated greater improvements in higher-order Theory of Mind and mentalization styles compared to the control group. While these findings are promising, disruptive behaviour was not assessed as an outcome, leaving it unclear whether gains in mentalizing translated into meaningful behavioural change. Moreover, the study did not examine the real-time application of mentalizing strategies during classroom incidents. As a result, the effectiveness of teacher-centred MBIs for managing disruptive behaviour, and the mechanisms through which they operate, remain insufficiently understood. Given how limited this evidence base is, the present findings are best read as exploratory and hypothesis-generating rather than as established support for classroom-wide application.

The mechanism through which teacher-centred MBIs work has not been clearly specified. A likely candidate is epistemic trust: the willingness to accept communicated knowledge as relevant and applicable to oneself [27,28]. When a teacher adopts a genuine mentalizing stance and attends to the mind behind the behaviour rather than the behaviour alone, the child is more likely to feel understood, which lowers the threat-focused vigilance that constrains mentalizing under stress and makes the child more able to take in what the teacher offers. This account connects directly to the biobehavioural switch model drawn on later (Section 4.2): being recognised reduces arousal, and reduced arousal restores the capacity to mentalize. Framing the predictions in these terms gives them a clearer mechanistic basis and links the present study to the developmental theory underlying MBT.

This study builds on Chelouche-Dwek et al. [29], one of the first investigations of teachers’ real-time use of MBIs in classrooms. Whereas this study established feasibility, the present study extends this work by (a) refining the MBI coding scheme to more closely align with MBT-C principles, particularly through the redefinition of Playing with reality to include playfulness; (b) adding audio recordings to enhance transcription fidelity; and (c) applying a cross-classified multilevel model to account for the nested data structure. These extensions strengthen its contribution to the evidence base on teacher-centred MBIs.

### 1.3. Present Study

To address these gaps, the present study examines the real-time application of teacher-centred MBIs in naturalistic classroom interactions—specifically during incidents of student disruptive behaviour. Real-time mentalizing, as used in this study, refers to the moment-to-moment application of mentalizing strategies by teachers as disruption unfolds—not in a structured session or curriculum, but in spontaneous response to immediate behaviour. This is different from programme-based MBI delivery, and it raises a distinct question: can mentalizing function effectively when it is unplanned, compressed, and embedded in the pressures of a live classroom? Because the study draws on a single specialist setting and an observational design, it is best understood as exploratory and hypothesis-generating: its aim is to identify candidate strategies and refine how they are operationalised, not to establish causal efficacy.

The following research questions were addressed:

- How does the application of MBIs by teachers during incidents of disruptive student behaviour predict the successful resolution of these incidents, and how does this vary by incident severity?
- What are the characteristics of these MBIs as enacted by teachers in the classroom?

To investigate these questions, a mixed-methods design was employed. Quantitatively, the study examined the efficacy of teachers using MBIs to manage disruptive behaviour. Qualitatively, it explored how these strategies were enacted in practice. This combined approach was intended both to assess the potential efficacy of teacher-centred MBIs and to generate practical insights to inform their refinement and implementation in schools.

Given these contextual factors and the broader evidence base on MBIs in schools [24], the following hypotheses were proposed:

**H1.**  *The use and combination of MBIs by teachers during incidents of disruptive behaviour would predict a higher likelihood of incident resolution*.

**H2.**  *A greater number of distinct MBIs used during incidents would be associated with a higher likelihood of resolution, suggesting additive effects consistent with findings in clinical contexts [30,31]*.

**H3.**  *The association between the number of MBIs used and incident resolution would be moderated by incident severity, with the direction of moderation remaining exploratory given mixed findings in MBT research [32]. The expectation that using more distinct MBIs would be associated with resolution (H2) is extrapolated from clinical psychotherapy and may not hold for brief, compressed classroom exchanges; it is therefore treated as a tentative, testable prediction rather than an established mechanism*.

## 2. Materials and Methods

### 2.1. Design

This study used a mixed-methods design to systematically examine the relationship between teachers’ use of MBIs and the resolution of disruptive behaviour in classrooms.

### 2.2. Setting

The study took place in a London-based alternative provision school. Many students at this school present with psychological profiles linked to mentalization difficulties, disruptive behaviour, and emotional dysregulation—including autism spectrum disorder (ASD), attention-deficit/hyperactivity disorder (ADHD), and trauma histories [33,34,35]. These characteristics increased both the likelihood of disruption and the potential relevance of MBIs. In addition, staff at the school receive regular trauma-informed training, making it more likely that teachers were already employing mentalizing-based strategies in their classroom practice [36]. The school provides an integrated educational and therapeutic provision, combining high-quality teaching methodologies with evidence-based practices drawn from contemporary Child and Adolescent Mental Health Services (CAMHS). Educational and therapeutic input is delivered within a systemic, multi-family-based framework designed to address unhelpful relational and behavioural patterns and support sustainable change.

### 2.3. Participants

Participants included students, teachers, and teaching assistants from two classrooms (Classroom 1 and Classroom 2) within a London-based alternative provision school.

Students were eligible if they were enrolled in either classroom during the observation period and informed consent was obtained from their legal guardians. Primary classroom teachers and teaching assistants assigned to these classrooms were included provided they gave informed consent. Any students or teaching staff who did not provide consent were excluded from observation and analysis.

Teaching staff at this school received ongoing trauma-informed and reflective practice training as part of the school’s integrated educational-therapeutic model—training that includes attention to mental states and emotional regulation. No formal MBI-specific programme was delivered before or during the observation period. Teacher responses were therefore coded as MBI-consistent rather than formally trained implementations; the study is best read as a naturalistic investigation of how MBI-aligned strategies emerge in everyday classroom practice.

Classroom 1 comprised five male students and one female student (mean age = 9.7 years). The primary classroom teacher was female with 9.5 years of teaching experience. Classroom 2 also consisted of five male students and one female student (mean age = 9.0 years). The primary classroom teacher was male with 3.5 years of teaching experience. Both classrooms included teaching assistants, maintaining a near 1:1 student-teacher ratio.

Across both classrooms, students presented with a range of neurodevelopmental and mental-health profiles, including autism spectrum conditions, ADHD, and histories of developmental trauma, and many met criteria for more than one.

In total, 12 students and 11 teachers participated in this study. Although the number of individual participants is modest, the primary unit of analysis was the incident part (N = 815), which constitutes a substantial observational dataset for examining moment-to-moment teacher responses. The cross-classified multilevel structure was chosen specifically to handle the small number of higher-level clustering units, partitioning variance attributable to students, teachers, and incidents as separate random effects rather than treating them as fixed.

For clarity and analytic consistency, all teaching staff (i.e., both classroom teachers and teaching assistants) are referred to collectively as teachers throughout the remainder of this paper. While their roles differ in terms of responsibility and training, both were actively involved in responding to disruptive behaviour incidents.

### 2.4. Measures

#### 2.4.1. Disruptive Behaviour Incident Model

Incidents of student disruptive behaviour (henceforth referred to as incidents) served as the primary unit of analysis. In total, 259 incidents were documented. To facilitate comparison with prior research, the definition and structural framework for incidents were adapted from Chelouche-Dwek et al. [29].

For the purposes of this study, an incident was defined as a discrete, observable period during which a student demonstrated behaviour that disrupted the classroom environment. Incidents were not treated as isolated events; rather, they were characterised by sequences of disruptive behaviour and corresponding teacher responses unfolding over time. Each incident was conceptualised as occurring in three sequential stages, representing the onset, core, and conclusion: (1) Pre-incident stage, (2) Incident stage, and (3) Post-incident stage. This tripartite structure provided a standardised method for identifying incident boundaries by anchoring each incident to observable behavioural markers: the onset of disruption (pre-incident), active disruption and teacher response (incident), and the resolution or continuation of behaviour (post-incident). This approach is consistent with event-based coding frameworks used in observational research on classroom behaviour [3].

To allow for a more fine-grained analysis, the incident stage was further segmented into smaller analytical units referred to as *incident parts*. An incident part was defined as a single occurrence of disruptive behaviour, the corresponding teacher response, and the observed outcome. Outcomes were classified as either *Resolution*—where the student ceased disruptive behaviour—or *Failure of resolution*—where disruptive behaviour continued. Segmenting incidents in this way enabled the identification of specific teacher responses immediately preceding successful or unsuccessful cessation of disruption, thereby allowing direct examination of whether teachers’ use of MBIs increased the likelihood of resolution.

There was no predetermined limit to the number of parts within an incident. Instead, parts were added sequentially until the post-incident stage was observed, with the number of parts varying according to the complexity and duration of each incident. By avoiding artificial cut-offs, this approach enabled a detailed and ecologically valid representation of each incident as it occurred in the classroom.

**Figure 1:**
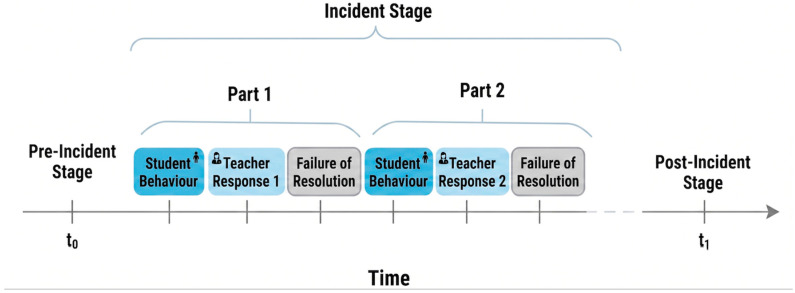
Disruptive behaviour incident model.

Figure 1 below provides a visual representation of the incident structure and its constituent parts.

#### 2.4.2. Coding Scheme for Mentalization-Based Interventions

To evaluate both the presence and number of MBIs teachers used during incidents, a structured coding scheme was developed and systematically applied to each teacher’s response.

This coding scheme was based on the framework proposed by Domon-Archambault et al. [37], who implemented a mentalization-based training programme for childcare workers. Their model was selected for its demonstrated applicability to non-clinical settings and its strong theoretical grounding in mentalization research [38,39,40]. Chelouche-Dwek et al. [29] subsequently adapted this framework for classroom contexts.

In the present study, their adaptation was further refined to more explicitly align with MBT for children (MBT-C) principles [22]. Notably, the category *Playing with reality* was redefined to reflect MBT-C’s play-centred approach by operationalising play in classroom contexts as playfulness and gentle humour, supported by research on playfulness as a pedagogical tool [41].

The final coding scheme included six distinct MBIs (see Table 1 for full definitions; see Supplementary Materials S2 for the full coding manual and operational definitions): (1) Mentalization—focusing on students’ mental states rather than their actions; (2) Validation—empathically acknowledging students’ experiences or reinforcing positive behaviour; (3) Down-regulation—calming strategies to reduce arousal and restore safety; (4) Playing with reality—using playfulness, humour, or reframing to shift perspective; (5) Collaboration—joint problem-solving or shared activity to promote agency; and (6) Scaffolding—developmentally appropriate support to reduce task difficulty.

Teacher responses within each incident part were independently coded for the presence (1) or absence (0) of MBIs based on this coding scheme. Multiple interventions could be coded within a single teacher response, allowing for the quantification of the number of distinct interventions used per response. Inter-rater reliability of the categorical scales was assessed using Cohen’s kappa, with agreement levels interpreted following Landis and Koch [42] benchmarks. Several of these strategies overlap with skilled general classroom management; what marks them as MBIs here is the explicit focus on the student’s mental states rather than on behaviour or task completion alone. Their discriminant validity relative to ordinary good teaching is considered further in the Discussion.

**Table 1:** Coding scheme of mentalization-based interventions.

**Mentalization**	Mentalization is central to a mentalization-based approach. It involves understanding and interpreting the student’s mental states (e.g., thoughts, emotions, desires), rather than focusing solely on their behaviour. It also involves the teacher sharing their own mental states to model self-**awareness and guide their interactions with the student.**
**Validation**	Validation involves accepting and acknowledging the student’s subjective emotional experience, regardless of its divergence from objective reality. It communicates to the student that their feelings and perceptions are understandable and legitimate. This intervention promotes a positive, hopeful, “can-do” attitude.
**Down-regulation**	Down-regulation involves the teacher’s efforts to reduce the student’s emotional arousal to a more manageable and tolerable level before engaging in mentalization. This may include soothing language, a soft tone of voice, non-threatening body language, or other calming techniques. This intervention fosters a sense of safety and security within the student.
**Playing with reality**	Based on the Playing with reality series [43,44,45,46], this intervention involves helping the student shift from a rigid, concrete perception of reality (psychic equivalence) to a more flexible one. The teacher may use playfulness or gentle humour to encourage exploration of alternative perspectives. This creates a safe environment for the student to consider that reality can be experienced and viewed in different ways.
**Collaboration**	Collaboration refers to the teacher involving the student in the co-construction of meaning and action. By positioning the student as an active partner in problem-solving or joint activity, this intervention fosters a sense of alliance between the teacher and the student and enhances the student’s sense of agency.
**Scaffolding**	Based on Vygotsky’s zone of proximal development [47], scaffolding involves tailoring strategies to the student’s current stage of cognitive and emotional development. The teacher provides the support necessary to enable the student to perform a task just beyond their current abilities, gradually reducing support as the student gains mastery.

This coding process enabled a systematic and theory-driven investigation of how specific mentalization-based strategies were deployed in classroom interactions, and how their presence—and number—related to incident outcomes.

#### 2.4.3. Incident Severity

Incident parts were also coded for the severity of disruptive behaviour. This approach allowed for precise analysis of whether the effectiveness of teacher-centred MBIs was moderated by incident severity.

The categorisation of incident severity was adapted from a model used at a UK primary school [48], which classified disruptive behaviour according to the degree of classroom disruption. This framework was selected because it closely resembled evidence-based systems for categorising disruptive behaviour in children [3], providing theoretical alignment.

To capture the full spectrum of behaviours relevant to disruptive episodes, indicators of emotional dysregulation were incorporated based on Freitag et al. [49], given the established link between dysregulation, mentalization, and disruptive behaviour [18,21,50].

The final adapted framework categorised behaviours into three severity levels—mild (1), moderate (2), and severe (3)—based on (a) the degree of classroom disruption and (b) the emotional dysregulation displayed (see Table 2). Each incident part was independently coded for severity according to this framework.

### 2.5. Procedure

#### 2.5.1. Observation Structure

Classroom observations were conducted over a 3-month period. Prior to data collection, both researchers completed joint calibration sessions during which they independently coded practice observation data and resolved discrepancies through discussion to ensure consistent application of the incident model and coding scheme. Formal observations then commenced, with two researchers independently observing classroom activity for a total of 96 h, divided evenly between two classrooms, with each researcher assigned to one classroom throughout the study.

#### 2.5.2. Introduction and Integration into the Classroom

To reduce the intrusiveness of the research environment, researchers were introduced to students by classroom teachers in a manner intended to normalise their presence. On the first day in each classroom, researchers focused on establishing rapport with students through informal interaction; formal observations began the following day. These steps were intended to reduce the likelihood that teachers’ or students’ behaviour would be altered by observation; however, observer and Hawthorne effects cannot be fully eliminated and may have influenced some interactions, and behaviour was not formally tested for stabilisation over the observation period (see Section 4.7).

#### 2.5.3. Ethical Considerations

The study received ethical approval from University College London (UCL) Ethics Committee. All participation was voluntary. Written informed consent was obtained from all participating teaching staff and teaching assistants. Written informed consent was obtained from the legal guardians of all participating students. All data were anonymised prior to analysis and handled in accordance with the UK General Data Protection Regulation (GDPR). Consent was obtained for every teacher and student in both classrooms, and no eligible participant declined to take part.

### 2.6. Data Collection

Data were collected using two concurrent methods: real-time written notes and continuous audio recording. When disruptive behaviour occurred, researchers recorded verbatim teacher–student dialogue alongside contextual information, including timing, antecedents, nonverbal behaviour, and teacher tone. Audio recordings supported full transcription of incidents and ensured data completeness when real-time note-taking was limited. At the end of each day of observation, researchers independently transcribed the disruptive behaviour incidents they had observed, using both written notes and audio recordings.

### 2.7. Data Analysis

#### 2.7.1. Coding and Inter-Rater Reliability

Once data collection was complete, two independent raters analysed transcripts of the observed incidents. Each incident part was coded for Resolution (1 = yes, 0 = no) and student behaviour severity (1 = mild, 2 = moderate, 3 = severe). Corresponding teacher responses were coded for the presence (1) or absence (0) of the six predefined MBIs. Worked examples of coded observation notes are provided in Supplementary Materials S3. The two researchers who carried out the observations also coded the transcripts. Observers and coders were therefore not independent, which could in principle inflate agreement; this is considered in Section 4.7. Consensus was reached only after independent coding and computation of kappa, so the reliability estimates were not affected by it, although we acknowledge that consensus may reinforce shared assumptions.

Inter-rater reliability was assessed using Cohen’s kappa for each of the six MBIs, and for incident resolution outcome (resolved vs. unresolved) and behaviour severity ratings (mild, moderate, severe). All kappa values exceeded the 0.75 threshold [42], indicating strong agreement across all coding dimensions. Following the initial reliability assessment, three researchers jointly reviewed incident parts and reached consensus on intervention coding through discussion. This consensus-coded dataset was used for all subsequent quantitative and qualitative analyses.

#### 2.7.2. Quantitative Analysis

All quantitative analyses were performed using SPSS Statistics (Version 30). The model syntax is provided in Supplementary Materials S1.

#### 2.7.3. Exploratory Chi-Square Analyses

Exploratory chi-square analyses examined whether individual MBIs and combinations of MBIs were associated with incident resolution. Due to violations of the independence assumption, resulting from multiple incident parts nested within incidents and repeated observations of teachers and students, findings were interpreted descriptively. These analyses were used to inform predictor selection for subsequent multilevel models, with infrequently used or non-informative interventions excluded to support model parsimony.

#### 2.7.4. Cross-Classified Multilevel Analyses

To evaluate whether the presence, combination, and number of MBIs predicted incident resolution—while accounting for non-independence of observations—a series of cross-classified multilevel logistic regression models (CCMM) [51,52] were conducted.

**Figure 2:**
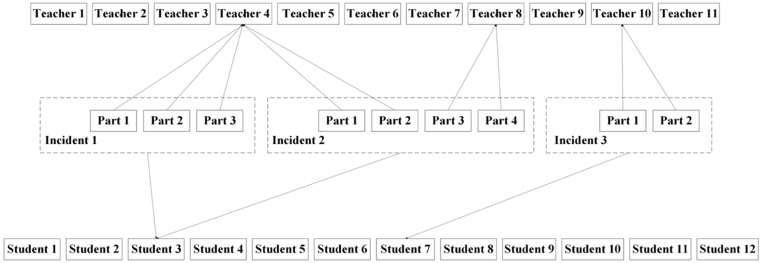
Cross-classified multilevel structure of the data.

Standard multilevel models assume strict hierarchical nesting, where each lower-level unit is linked to exactly one higher-level unit. In this dataset, that assumption did not hold. Incident parts were nested within broader disruptive behaviour incidents. Each part was associated with both a student and a teacher, but students and teachers were not uniquely paired or nested within each other. Instead, they appeared in various combinations across incidents. This created a cross-classified structure in which incident parts were simultaneously grouped by student and teacher, but neither group was nested within the other. Figure 2 illustrates the cross-classified multilevel structure of the data. Although the structure is cross-classified, students were also partially nested within teachers, because each student was taught mainly, though not only, by the staff in their own classroom; the model takes this partial nesting into account.

Given this complexity, CCMM was selected as the most appropriate analytic approach. CCMM extends standard multilevel models by incorporating random effects for each crossed grouping factor, allowing the model to partition variance attributable to students, teachers, and incidents. Because only 11 teachers and 12 students contributed data, power to estimate higher-level variance components was limited; the cross-classified structure was used to partition this variance appropriately rather than to ignore it, and variance components were also estimated in null models containing no predictors.

#### 2.7.5. Qualitative Analysis

A qualitative analysis was conducted to examine how MBIs were enacted by teachers during disruptive incidents, providing contextual insight into strategy use beyond categorical coding.

The analysis followed Braun and Clarke [53] six-phase approach to thematic analysis. Given that data were pre-coded for six MBIs, a primarily deductive and semantic approach was adopted, while allowing inductive insights to emerge regarding tone, timing, and delivery. The dataset comprised the consensus-coded incident parts and was analysed in ATLAS.ti (version 25.0.1).

Thematic development focused on identifying patterns in how interventions were enacted and refined through iterative comparison within each intervention type.

To support analytic integrity, an independent researcher reviewed 25% of the incident parts, following O’Connor and Joffe [54] guidance. Discrepancies were resolved through discussion, and revisions were applied across the dataset.

Both researchers were trained in mentalization theory, which inevitably shaped what they noticed, recorded, and coded, and their presence in the classrooms, while enriching contextual understanding, introduced a risk of confirmatory interpretation. To mitigate this, all coding was carried out after observations had ended; the coding categories were operationally defined in advance; inter-rater reliability was formally assessed; and discrepancies were resolved through structured discussion. The interpretations should therefore be read as theory-informed rather than theory-neutral.

## 3. Results

A total of 259 incidents and 815 incident parts were recorded.

### 3.1. Quantitative Results

#### 3.1.1. Reliability of the Mentalization-Based Interventions Coding Scheme

The reliability analysis yielded high Cohen’s kappa coefficients for all intervention categories, exceeding the benchmark of 0.75. These results indicate strong inter-coder agreement beyond chance, supporting both the reliability of the coding scheme and the validity of the data. Each intervention demonstrated statistically significant kappa values, as outlined below:

*Mentalization:* A perfect kappa coefficient of 1.00 was achieved (*p* < 0.001), indicating that coders reliably identified instances where teachers focused on students’ mental states as opposed to their actions.
*Validation*: The intervention yielded a kappa coefficient of 0.76 (*p* < 0.001), demonstrating strong agreement in identifying instances where teachers promoted a hopeful, can-do attitude or validated students’ subjective experiences.
*Down-regulation:* This intervention achieved a kappa score of 0.92 (*p* < 0.001), indicating that coders consistently recognised strategies focused on fostering a sense of safety and security.
*Playing with reality:* A perfect kappa coefficient of 1.00 was achieved (*p* < 0.001), demonstrating complete agreement on instances where teachers helped students shift from a concrete perception of reality to a more flexible one.
*Collaboration:* This intervention yielded a kappa coefficient of 0.84 (*p* < 0.001), indicating reliable identification of instances where teachers used joint problem-solving or activities to enhance students’ sense of agency.
*Scaffolding:* Another perfect kappa score of 1.00 (*p* < 0.001) confirmed consistent agreement on identifying instances where teachers implemented developmentally appropriate scaffolding strategies.

These robust reliability measures validate the methodological rigour of the study and provide strong support for the accuracy of MBI identification within the dataset.

#### 3.1.2. Preliminary Analyses

Chi-square tests of association were conducted to examine whether MBIs were co-implemented by teachers during incidents more often than expected by chance. Several statistically significant associations emerged, with effect sizes ranging from small to medium. Notably, Mentalization was significantly associated with Validation, χ^2^ (1) = 44.077, *p* < 0.001, φ = 0.233; Down-regulation, χ^2^ (1) = 41.336, *p* < 0.001, φ = 0.225; and Collaboration, χ^2^ (1) = 14.270, *p* < 0.001, φ = 0.132. Additional significant associations were found between Validation and Down-regulation, χ^2^ (1) = 20.230, *p* < 0.001, φ = 0.158; Collaboration and Scaffolding, χ^2^ (1) = 12.224, *p* < 0.001, φ = 0.122; and Collaboration and Playing with reality, χ^2^ (1) = 4.968, *p* = 0.026, φ = 0.078. These findings suggest that these intervention pairs were implemented together more frequently than expected by chance. No other intervention pairs showed significant associations.

Overall, these findings indicate statistically significant small-to-moderate associations between several intervention pairs, highlighting combinations commonly used by teachers to manage disruptive behaviour. Table 3 presents the chi-square results for all intervention pairs.

Additional chi-square tests were conducted to examine whether the use of individual interventions predicted incident resolution. As the independence assumption was violated, these results are interpreted as preliminary associations rather than definitive predictive effects. Table 4 presents the contingency tables for each intervention, showing frequency distributions across intervention use (used vs. not used) and incident resolution outcomes (resolved vs. not resolved), along with chi-square results. Accordingly, we limit the interpretation of these chi-square results and rely on the multilevel models for inference.

Given that Scaffolding did not show a significant association and was used relatively infrequently (*n* = 18), it was excluded from subsequent analyses to support statistical robustness and model parsimony. Because Scaffolding was not entered into the multilevel models, its lack of association was tested only descriptively and should be interpreted with caution rather than as a formally estimated null effect.

#### 3.1.3. Cross-Classified Multilevel Analyses

Random effects for teacher, student, and incident identifiers were small and non-significant across both models, indicating no detectable clustering by these factors. With only 11 teachers and 12 students, however, the power to estimate these variances was limited, so this reflects an absence of detectable clustering rather than firm evidence that teacher- or student-level effects are absent. As is standard practice, variance components were also non-significant in null models containing no predictors. Results for both models are presented in Table 5.

Model 1 results revealed that the use of all interventions except Collaboration was associated with significantly higher odds of resolution:

Mentalization: Using Mentalization was associated with significantly higher odds of resolution, OR = 5.183, 95% CI [3.448, 7.791], *p* < 0.001, suggesting that shifting the focus from students’ actions to the underlying mental states was associated with resolution.

Validation: Using Validation was associated with significantly higher odds of resolution, OR = 2.368, 95% CI [1.507, 3.723], *p* < 0.001, suggesting that validating students’ subjective experiences was associated with resolution.

Down-regulation: The use of Down-regulation was also associated with a significant increase in the odds of resolution, OR = 3.753, 95% CI [2.428, 5.801], *p* < 0.001, suggesting that calming strategies to foster safety and security were associated with resolution.

Playing with reality: Using Playing with reality was associated with significantly higher odds of resolution, OR = 3.411, 95% CI [2.088, 5.574], *p* < 0.001, indicating that encouraging flexible perspectives through playfulness or humour was associated with resolution.

Collaboration: Using Collaboration did not significantly predict resolution, OR = 1.544, 95% CI [0.790, 3.018], *p* = 0.203, suggesting that joint activity or problem-solving did not contribute significantly. To aid interpretation, these odds ratios correspond to sizeable differences in model-predicted probability of resolution for a typical (median) incident: from a baseline of roughly 10% when an intervention is absent to about 37% for Mentalization, 30% for Down-regulation, 28% for Playing with reality, and 21% for Validation when that intervention is present. These are illustrative marginal predictions at the median random effect and should be read alongside the wide confidence intervals reported above.

In Model 2, when interaction effects were accounted for, Validation was no longer a significant predictor (OR = 1.753, 95% CI [0.705, 4.266], *p* = 0.230), suggesting that this intervention may not function as a stand-alone strategy. In contrast, Mentalization (OR = 3.201, 95% CI [1.659, 6.177], *p* < 0.001), Down-regulation (OR = 3.603, 95% CI [1.787, 7.264], *p* < 0.001), and Playing with reality (OR = 2.665, 95% CI [1.250, 5.682], *p* = 0.011) remained significant predictors. Collaboration remained non-significant (OR = 1.290, 95% CI [0.265, 6.265], *p* = 0.752). Because Model 2 contains interaction terms, these lower-order coefficients are conditional effects (the association of each intervention when the others are absent) and should not be read as average main effects.

Most two-way interactions were not significant (see Table 6), suggesting that intervention combinations generally did not provide additional predictive value beyond their individual effects. The exception was the interaction between Mentalization and Playing with reality, OR = 3.060, 95% CI [1.042, 8.988], *p* = 0.042. Although this combination was not observed more frequently than expected by chance (see Table 4), shifting focus to mental states while simultaneously encouraging flexible perspectives through humour or playfulness appeared to have a synergistic association, enhancing the likelihood of resolution beyond their individual contributions. This interaction was one of ten interaction tests and was significant only at the uncorrected level (*p* = 0.042); it would not survive correction for multiple comparisons and is reported as exploratory and hypothesis-generating. Several interaction estimates also carried very wide confidence intervals (for example, Mentalization × Collaboration, 95% CI [0.562, 13.965]), indicating considerable instability, so they should be interpreted with caution.

In summary, results suggest that Mentalization, Down-regulation, and Playing with reality predicted a higher likelihood of resolution, whereas Collaboration did not. Validation was significant only when interaction effects were not included. Only the combination of Mentalization and Playing with reality demonstrated a synergistic effect.

#### 3.1.4. Additive Effect of Interventions on Incident Resolution and Moderation by Severity

As in Models 1 and 2, random effects were small and non-significant—consistent with MBI use, rather than person-level characteristics, being associated with the observed resolution patterns, though the limited power to estimate higher-level variance means person-level effects cannot be ruled out. Results for Model 3 are presented in Table 6.

Model 3 showed that, for each additional distinct MBI used, the odds of resolution were higher by a factor of 3.293, 95% CI [2.690, 4.029], *p* < 0.001. Because Model 3 includes the Number × Severity interaction terms, this figure is the additive effect estimated at the reference (mild) severity level rather than an average main effect (as noted for Model 2). This indicates a strong additive association: the more interventions applied, the greater the likelihood of resolution. To aid interpretation, this corresponds to a rise in the model-predicted probability of resolution for a typical (median) incident at the reference (mild) severity level from roughly 10% with no MBI to about 27% with one, 55% with two, and 80% with three distinct MBIs. Because incident parts accumulated until an incident was resolved, however, longer or more complex incidents tended to generate both more parts and more interventions, so this additive association may partly reflect incident duration or complexity rather than a dose effect of the interventions themselves (see Section 4.7).

Compared with incidents of mild severity, incidents of moderate severity did not differ significantly in odds of resolution (OR = 0.859, 95% CI [0.531, 1.390], *p* = 0.651), suggesting similar likelihoods of resolution between mild and moderate severity. Severe incidents had significantly lower odds of resolution than mild incidents (OR = 0.584, 95% CI [0.344, 0.992], *p* = 0.047), indicating that severe incidents were less likely to be resolved.

The interaction terms between intervention number and incident severity were not significant (OR = 1.075, 95% CI [0.655, 1.765], *p* = 0.775 for moderate; OR = 1.449, 95% CI [0.880, 2.357], *p* = 0.147 for severe), indicating that severity did not moderate the relationship between intervention number and resolution. Because both interactions point estimates were greater than 1, the additive slope implied at moderate and severe levels was, if anything, marginally steeper than at mild severity, so the reference-level estimate reported above is a conservative expression of the additive effect rather than an overstatement of it. In other words, the association between using more interventions and higher odds of resolution was consistent across mild, moderate, and severe incidents.

Overall, these findings suggest that employing a greater number of distinct MBIs during disruptive incidents is associated with a higher likelihood of resolution, and that this additive association appeared comparable across levels of incident severity.

### 3.2. Qualitative Results

**Figure 3:**
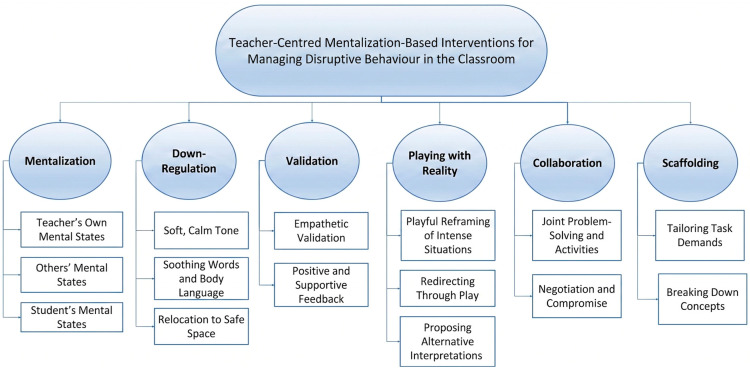
Map of thematic patterns by mentalization-based intervention.

The thematic analysis of classroom dialogue during disruptive behaviour revealed recurrent patterns in how each MBI was enacted by teachers. A thematic map of these patterns is presented in Figure 3.

The following sections present these patterns, ordered by frequency from most to least frequently coded. Anonymised excerpts from classroom dialogue are included to illustrate and substantiate the themes. Although quantifying terms such as frequently or prominent are used to convey general trends, these are intended as contextual indicators rather than precise measures of prevalence.

#### 3.2.1. Mentalization

A prominent strategy for enacting Mentalization involved teachers modelling reflective thinking by sharing their own mental states during disruptive episodes. This was often achieved by explicitly communicating their thoughts, perceptions, or interpretations of the behaviour:

“I can’t read your mind, right? From what I saw you just got up and started running outside.”

[Teacher, Male]

Teachers also expressed their own emotions in response to behaviour:

“Can you please pull your chair up? I’m scared you’ll hurt yourself.”

[Teaching Assistant, Female]

Another common approach was encouraging students to consider others’ mental states. For example, when a student turned off the classroom lights, a teacher prompted reflection on peers’ preferences, leading to resolution:

“What about other people? Did you ask other people if they wanted it like this?”

[Teacher, Male]

Teachers also demonstrated a mentalization focus by inquiring about the mental states underlying disruptive behaviour, using open-ended prompts such as “What’s wrong?”, “Can you please use your words?”, or “What’s going on?”

In some cases, teachers used more close-ended questions to tentatively communicate interpretations of students’ mental states, linking behaviour to possible feelings or needs:

“Is it the sound or the laptop that is frustrating you? Do you want to sit in the other class for a bit?”

[Teaching Assistant, Female]

#### 3.2.2. Down-Regulation

The most prominent Down-regulation strategy involved using a soft, calm tone and speaking slowly. This gentle delivery was often paired with soothing language, such as “That’s okay,” “It’s okay,” or “Okay” at the start or end of responses.

Teachers also frequently employed calming body language, including kneeling or crouching to the student’s eye level, sitting beside them, or moving slowly and gently during incidents. In some cases, teachers provided students with the option to change their environment, such as retreating to the sensory room or moving to a quieter location away from triggers. This was exemplified with statements such as “Let’s step out for a minute” or “Do you want to go somewhere where it is nice and quiet?”

#### 3.2.3. Validation

The most prominent Validation strategy involved empathetically affirming students’ subjective experiences. This was typically achieved by acknowledging the legitimacy of the student’s perspective and recognising the complexity or difficulty of the situation. For example, in response to dysregulation triggered by a peer’s behaviour:

“I’m sorry, that’s not very kind, is it? He should’ve asked.”

[Teacher, Male]

Another prominent Validation strategy involved providing affirmative and supportive feedback, expressed through positive language (e.g., “Thank you,” “Well done,” “Good job”) and an encouraging tone. Teachers often used this strategy to positively reinforce the de-escalation of disruptive behaviour; when behaviour had reduced in severity but not yet fully resolved. Such reinforcement frequently contributed to complete resolution.

For example, when a student was dysregulated and engaging in negative self-talk, a teacher responded by writing down and reading aloud positive statements about the student:

“[Student Name] is smart, funny, kind, and nice. He makes the classroom a happier place. It’s happier when you’re here—well done!”

[Teacher, Male]

Affirmative feedback was also frequently provided immediately after incidents were resolved, drawing attention to improvements in emotional regulation or behaviour.

#### 3.2.4. Playing with Reality

The most common way teachers enacted Playing with reality was during intense situations, using playfulness or gentle humour to break tension and reframe the situation as light-hearted. Teachers typically adopted an amused, dramatic, or upbeat tone. For example, when a student was hitting objects, a teacher reframed the situation as a playful contest:

“Unfortunately, a table is always going to win, so don’t square up.”

[Teacher, Female]

Another common use of this strategy involved redirecting students toward playful activities, inviting them into an altered, more positive reality. For instance, when a student was ripping papers off the wall, teaching assistants repurposed the paper into a playful challenge, successfully diverting attention:

“I was wondering if you could see how many times you can fold this paper in half? I think the record is seven times!”

[Teaching Assistant, Female]

Less frequently, Playing with reality was applied in subtler ways without overt humour or playfulness. In these instances, teachers offered tentative, positively framed suggestions encouraging students to consider alternative perspectives. For example, when a student became upset because peers touched his artwork (believing they intended to damage it), a teaching assistant offered a reinterpretation that led to resolution:

“I think they’re just using your nice design for their world. That’s nice!”

[Teaching Assistant, Female]

#### 3.2.5. Collaboration

The most prominent Collaboration strategy involved inviting students to participate in joint activities or problem-solving with the teacher, emphasising partnership rather than authority. This was often conveyed by using language such as “let’s” instead of “you must”:

“Let’s make a plan for the day together!”

[Teacher, Male]

Another common approach was the use of we language (e.g., “We’ll do this together,” “We’re going to get this cleaned up”), which conveyed shared ownership of the task or process.

A less common Collaboration strategy involved negotiation and compromise, particularly during incidents of off-task behaviour or refusal to follow instructions. For example:

“Once you’ve finished organising your things I’ll let you have laptop time. Is that a fair deal?”

[Teacher, Female]

#### 3.2.6. Scaffolding

Scaffolding was often used when students were off-task, inattentive, or expressed difficulty understanding the task. This strategy typically involved reducing task complexity so students could build confidence before attempting full requirements. Examples included providing sentence starters or visual cues, breaking the task into smaller components, or prompting the student to attempt only part of the work.

This strategy was also used to make abstract concepts or difficult instructions more accessible. Teachers accomplished this by asking clarifying questions such as “What don’t you understand?” and then breaking the material down into simpler, more manageable steps.

#### 3.2.7. When Interventions Did Not Resolve Incidents

Not every MBI resolved the incident in which it was used, and the same technique that resolved one incident sometimes failed in another. The qualitative material suggested that what differed was less the choice of strategy than its timing, the student’s level of arousal, and the manner of delivery. Mentalizing prompts that invited reflection (for example, “What’s going on for you?”) tended to fall flat when a student was still highly aroused, and landed better once Down-regulation had lowered arousal first. Playful reframing that defused one situation could read as dismissive in another, particularly when a student felt their distress was being made light of. These patterns are consistent with the biobehavioural switch model and with the synergy observed between Mentalization and Playing with reality, and they suggest that effectiveness depends on attuning the intervention to the moment rather than on the intervention type alone. Two brief examples illustrate this.

For example, when a student returned to the room, screamed, and knocked objects off his desk, a reflective prompt delivered in a calm tone did not de-escalate the situation:

“Can you please use your words instead of your hands? Please stop. Why are you doing this?” [Teacher] The student continued to scream, and the incident remained unresolved.

In another incident, a playful response escalated rather than defused the situation: when a student knocked down a tower of cards the teacher was building, the teacher replied in a light, animated tone,

“I had a feeling that was going to happen.” [Teacher] The student then shouted, “Get off my desk!”, and the incident escalated.

## 4. Discussion

### 4.1. Overview

The present mixed-methods study examined the real-time application and correlates of teacher-centred mentalization-based interventions (MBIs) in managing disruptive classroom behaviour. Quantitative analyses identified specific MBIs, and one combination, that were associated with a higher likelihood of incident resolution, while others were less effective (H1). Using a greater number of distinct MBIs during an incident was consistently associated with a higher likelihood of resolution, demonstrating robust additive effects (H2) that were maintained across incident severity levels (H3). Qualitative analyses complemented these findings by illuminating how teachers flexibly enacted MBIs in practice, highlighting tone, timing, and style. Together, the findings position teacher-centred MBIs as promising candidate strategies that advance both theoretical understanding and practical guidance for managing disruptive behaviour. The qualitative and quantitative strands converge in places: the two forms of Validation identified qualitatively help explain why Validation was significant on its own but not alongside other interventions, and the playful form of Playing with reality offers a candidate mechanism for its synergy with Mentalization.

### 4.2. Managing Disruptive Behaviour Through Mentalizing and Emotional Regulation

Mentalization emerged as one of the strongest correlates of resolution, supporting theoretical and empirical work linking disruptive behaviour and emotional dysregulation to breakdowns in mentalization [18,55]. When teachers stimulated students’ mentalizing capacities—through perspective-taking, curiosity about inner states, or sharing mental states—incidents were more likely to resolve. Notably, teachers appeared to stimulate mentalization precisely at moments of breakdown, echoing a core paradox in mentalization-based treatment (MBT) [12].

Consistent with MBT theory, Down-regulation also significantly associated with resolution. According to the biobehavioural switch model, heightened arousal constrains reflective mentalization, rendering regulatory strategies essential [15,56]. Teachers who prioritised calming strategies—such as lowering tone, offering containment, or changing physical context—were more likely to resolve incidents, echoing findings by Chelouche-Dwek et al. [29]. These results suggest that teachers, like MBT therapists, must continuously balance reflection and regulation, albeit within more compressed and demanding conditions [22].

These findings extend MBT theory by demonstrating that pacing interventions to arousal is relevant beyond clinical settings. Mentalization appears most effective when arousal is lower, whereas Down-regulation becomes critical when arousal is high. Future research should directly test whether student arousal or incident severity moderates these effects, and whether moderately severe incidents represent a window in which combining strategies is particularly effective.

### 4.3. Empathetic Validation and Positive Reinforcement: Clarifying the Role of Validation

Another MBT intervention which, according to MBT theory, is particularly effective in instances of high emotional arousal is empathetic validation—showing empathy and acknowledging patients’ subjective experiences [22]. Chelouche-Dwek et al. [29] found no independent effect of Validation on incident resolution and argued that it was conceptually subsumed under other strategies. Because we found an independent effect that disappeared once interactions were included, a more nuanced interpretation is warranted: Validation may function less as a stand-alone strategy and more as a supportive precursor that enhances the effectiveness of subsequent MBIs.

This interpretation aligns with MBT, where empathetic validation forms part of the basic mentalizing stance [22]. In MBT and MBT-C, sessions typically begin with empathy and validation to establish emotional attunement with the patient [22,57]. The classroom data suggest a similar process, but with a key difference: whereas therapists sustain a validating stance throughout an entire session, teachers tended to use Validation episodically, in response to disruptive incidents.

A further contribution lies in showing that Validation is not a single construct but takes two distinct forms in classrooms: (a) empathetic validation of students’ subjective experience (closely aligned with MBT principles [22]) and (b) validation of students’ progress in regulation or behaviour management (more aligned with behaviourist reinforcement approaches [58]). In MBT, progress-oriented validation typically refers to long-term therapeutic progress and is subsumed under supportive interventions [22,59]. In classrooms, it appeared to operate as immediate positive reinforcement; for example, praising a student for regulating or re-engaging with work. Empathetic validation likely operated as a precursor that paved the way for other MBIs, while progress-oriented validation may have contributed more directly to incident resolution, behavioural de-escalation. Because both were coded together, their distinct effects were likely obscured.

This study therefore makes an important methodological contribution by arguing that Validation should be coded in classrooms as these two separate constructs. Future research should examine these roles more explicitly; for example, by using sequential analysis [60] to test whether empathetic validation typically precedes other MBIs. At a theoretical level, these findings highlight a broader opportunity to integrate behavioural reinforcement and mentalization-based approaches.

### 4.4. Translating Play from MBT-C to the Classroom Context

The present study found that Playing with reality was significantly associated with incident resolution, whereas Chelouche-Dwek et al. [29] reported no such effect. One likely explanation lies in differences in operationalisation. Here, Playing with reality explicitly included teachers’ use of playfulness and gentle humour to reframe situations. By integrating playfulness, the intervention more closely resembled the play-centred focus of MBT-C [22] and drew on foundational work linking pretend play to the development of mentalization [43,44,45,46].

In practice, teachers appeared to use Playing with reality most often during intense situations. Rather than the extended play sequences typical of MBT-C [22], teachers employed brief playful reframings that invited students to consider alternative perspectives of reality, helping to restore mentalizing capacities and disengage from disruptive behaviour. Where structured play is not feasible, playfulness may act as a functional proxy for pretend play, woven into real-time teacher–student interactions. This interpretation resonates with literature on creative pedagogies, where playfulness—or playful learning—is used to reframe learning as dynamic and engaging [41,61,62]. Importantly, this literature distinguishes between guided play, initiated by teachers, and self-directed play, initiated by students and supported by teachers [63]. Extending this pedagogical lens to behaviour management suggests that Playing with reality may also operate in both guided and self-directed forms—an important hypothesis for future research.

Notably, Playing with reality was also the only intervention that, when combined with Mentalization, produced a synergistic effect on resolution. Qualitative analyses suggested that Playing with reality was enacted in two ways: (a) overtly playful reframing and (b) non-playful but positive proposals of alternative interpretations of reality. The synergy may therefore have two explanations. First, in its non-playful form, Playing with reality may complement Mentalization by jointly scaffolding perspective-taking, consistent with its role in MBT-C [22]. Alternatively, when combined with playful reframing, the play element may reduce the cognitive–emotional load of reflective work, rendering mentalization more accessible during emotionally charged moments, in line with the biobehavioural switch model [15,56]. This interpretation echoes child psychotherapy findings that play provides a safe medium for processing difficult emotions without overwhelming the child [64,65]. Humour is not uniformly benign, however. Playfulness that is mistimed or poorly attuned, for example at peak arousal or when a student reads it as mockery, can come across as dismissive and escalate rather than defuse an incident. Playing with reality is therefore best understood as a skill that depends on attunement to timing and arousal, which carries a clear training implication and points to future work on when playfulness helps and when it backfires. Taken together, these findings highlight playfulness as a novel pathway for embedding MBT-C play-based principles in classrooms, while underscoring the need for more precise coding. This study argues that Playing with reality should be coded along two dimensions in classrooms: (1) playful vs. non-playful applications and (2) guided (teacher-initiated) vs. self-directed (student-initiated) playfulness. A valuable next step would be to test these distinctions and their respective contributions, both independently and in combination with Mentalization.

### 4.5. Evaluating the Classroom Application of Collaboration and Scaffolding

This study also identified two MBIs that were not associated with resolution in our analyses—Collaboration and Scaffolding—diverging from Chelouche-Dwek et al. [29]. Far from being disappointing, these null findings are instructive: they highlight how easily MBT-informed strategies can drift away from their theoretical roots when adapted to classrooms. In Domon-Archambault et al. [37], Collaboration referred to co-constructing children’s mentalizing skills, while Scaffolding involved structuring challenges to stimulate mentalization. In our adaptation, however, both strategies were primarily applied toward classroom work rather than mentalizing skills. Although these adaptations reflected the realities of classroom life, they may have also diluted the mentalization focus central to these strategies’ effectiveness. These two strategies should be treated differently, however. Collaboration was retained as a predictor in the multilevel models and showed no significant association, whereas Scaffolding was used too infrequently (*n* = 18) to enter the models and was assessed only with descriptive chi-square tests. Scaffolding’s null status is therefore not a formally tested result and we interpret it cautiously.

Rather than discarding these strategies, these findings point to a re-operationalisation grounded more firmly in mentalization theory. Collaboration often involved the use of “we” language to emphasise shared ownership of tasks (e.g., “We’ll do this together”), echoing MBT’s “we-mode”, which fosters joint attention and shared intentionality [66]. Yet while MBT therapists use this stance to explore ruptures in mentalizing, teachers largely applied it to secure task engagement. A promising next step would be to re-operationalise Collaboration as explicitly mentalization-focused: using “we-mode” to explore the mental states underlying disengagement, rather than merely redirecting behaviour.

Scaffolding, by contrast, may require a different kind of rethinking. In MBT-C, scaffolding extends beyond individual sessions, involving parents and the child’s wider social network to create a mentalization-supportive environment [22]. Rather than a moment-to-moment classroom tool, Scaffolding may hold greater promise when embedded within school-wide approaches, where mentalizing principles are integrated into the broader school climate [67].

Alternatively, these null findings may reflect a need to reframe rather than re-operationalise these strategies. Qualitative analyses suggested that Collaboration was typically used to structure routines (e.g., “Let’s make a plan for the day”), while Scaffolding targeted off-task behaviour stemming from inattention or difficulty understanding material. Rather than resolving incidents, such uses position these strategies as preventive supports, shaping conditions that reduce frustration and disengagement before escalating to disruptive behaviour. Evaluating these strategies’ preventive value represents an important next step.

Taken together, the null findings underscore a key challenge of translational work: adapting MBT strategies for feasibility in classrooms without sacrificing theoretical fidelity. Rather than discarding Collaboration and Scaffolding, these results invite a strategic rethink: testing more theory-anchored operationalisations, situating Scaffolding within systemic approaches, and exploring their potential as proactive rather than reactive interventions.

A broader interpretive question is whether these strategies capture mentalization specifically or simply reflect high-quality teaching. Several MBIs overlap with skilled classroom management and responsive pedagogy, and strategies such as Validation, Collaboration, Scaffolding, and Down-regulation are recognised components of evidence-based teaching irrespective of mentalization theory, and the present design cannot fully separate the two. These categories were not drawn from generic pedagogy: they were operationalised as mentalization strategies within mentalization-based training frameworks [37] and coded as such in our earlier classroom study [29], so their overlap with effective teaching reflects shared observable behaviour rather than a shared theoretical origin. What distinguishes the coding used here is its explicit anchoring in the student’s mental states rather than in behaviour or task completion alone; the distinctive theoretical contribution of MBIs is not the strategies themselves but the sustained, explicit focus on the mental states underlying behaviour, and the associated claim that these strategies work by restoring mentalizing rather than through behavioural control alone; even so, establishing discriminant validity will require comparison conditions or contrasts with established classroom-management frameworks, coding the explicit mental-state focus separately from the surface strategy, and testing whether this mentalizing component predicts resolution over and above generic responsive pedagogy. We therefore frame these strategies as mentalization-informed teaching rather than as a wholly distinct practice.

### 4.6. Additive Effects of Teacher-Centred MBIs Across Incident Severity

A central finding was the additive benefit of drawing on a broad repertoire of MBIs. Incidents were more likely to be resolved when teachers layered strategies, regardless of severity. This mirrors MBT findings showing that combining techniques strengthens alliance and mentalizing [30,31], suggesting that breadth of response may itself represent a key professional skill, with clear implications for teacher training.

Additive effects appeared to arise from accumulation rather than synergy, with only one synergistic pairing identified. That said, our analysis focused on co-occurrence, and synergy may also depend on sequencing; for example, Validation laying the groundwork for Mentalization. Sequential coding [60] represents a valuable next step. Importantly, additive benefits were stable across severity levels. One interpretation is that teacher-centred MBIs act on a shared underlying mechanism—resolving breakdowns in mentalizing—regardless of severity [12]. Two cautions temper the additive finding. First, because incident parts accumulated until resolution, longer or more difficult incidents tended to generate both more parts and more interventions, so the association may partly reflect incident duration or complexity rather than a dose effect (see Section 4.7). Second, the direction of association cannot be established: teachers may have layered more interventions while a student was already beginning to settle, rather than the interventions producing the settling. A greater number of interventions may also index teacher effort or attentiveness rather than better practice, so breadth should not be equated with quality; attunement and timing are likely to matter as much as the number of strategies used.

### 4.7. Limitations

Several limitations should be noted. The study was conducted in two classrooms within one London alternative provision school, which limits generalisation to mainstream or other specialist settings. This is an inherent feature of naturalistic, in-depth observational work rather than a sampling error: the depth of coding (815 incident parts, each coded across seven dimensions) required extended immersion in a stable context. The person-level sample (12 students, 11 teachers) is modest, but the primary unit of analysis—the incident part—yielded a dataset adequate for the multilevel analyses used; with only 11 teachers and 12 students, the non-significant random effects indicate no detectable clustering rather than confirming the absence of student- or teacher-level confounding, given the limited power to estimate these variances. The observational design precludes causal inference; observed associations may reflect unmeasured factors such as classroom climate, concurrent behaviour management strategies, teachers’ interpersonal style, or a general responsiveness or relational warmth that operates independently of mentalization [3,68]. Researcher expectations may also have influenced observation and coding, though systematic coding and high inter-rater reliability across all dimensions reduced this risk. The binary outcome measure (resolved vs. unresolved) likely oversimplifies MBI effects. Future research should examine more nuanced outcomes, such as the degree of de-escalation, maintenance of resolution, and students’ socio-emotional development.

The study took place in a single specialist alternative-provision school whose embedded trauma-informed, reflective-practice culture is likely to have raised the baseline use of MBI-aligned strategies, limiting generalisation to mainstream schools. The sample was also narrow (roughly five boys to every girl; ages around nine), so the findings may not extend to girls, given sex differences in externalising behaviour and emotion regulation, to other ages, or to other cultural contexts. The near one-to-one teacher-to-student ratio, unavailable in larger mainstream classes, means the findings show what MBIs can achieve under highly favourable rather than typical conditions.

Several methodological constraints warrant caution. Familiarisation was intended to reduce reactivity, but observer and Hawthorne effects cannot be ruled out [69], and behaviour was not tested for stabilisation. With notes and audio but no video, non-verbal delivery central to Down-regulation and Playing with reality was captured only through observer notes. Because the two observers also coded the transcripts, the high inter-rater agreement, including a perfect kappa for Mentalization, Playing with reality, and Scaffolding, may partly reflect shared framing; consensus followed kappa computation, so it did not affect the estimates but may have reinforced shared assumptions. The severity framework was adapted for this study and is not psychometrically validated.

Finally, several features limit causal interpretation. The additive association may partly reflect incident duration or complexity, since longer incidents generated more parts and more opportunities for intervention, and its direction cannot be established, as teachers may have layered interventions while a student was already settling. Analysing all staff as “teachers” overlooks differences between teachers and teaching assistants in training, role, and authority relevant to the paper’s training implications. Unmeasured factors, including teacher personality, relational quality, classroom climate, and the marked experience gap between the two lead teachers (9.5 versus 3.5 years), may also contribute; teacher identity was modelled as a random effect, which partially accounts for but does not eliminate this confound. Finally, with only 11 teachers and 12 students and no a priori power calculation, power to estimate higher-level variance was low, so the non-significant random effects indicate no detectable clustering rather than confirmed absence; multiple comparisons raise the risk of Type I error, and rare interventions raise the risk of Type II error.

### 4.8. Implications and Future Directions

This study is among the first to systematically analyse the real-time use of teacher-centred MBIs in naturalistic classroom settings. The findings strengthen the limited evidence base for teacher-centred MBIs [26] and extend a growing body of work exploring MBIs in schools and other non-clinical contexts [24].

More than a proof of concept, this study positions MBIs as promising candidate strategies, while also clarifying how their operationalisation should be refined before scaling up. Once these refinements are established, the next step will be to test the causal efficacy of these strategies through rigorous, multi-school intervention trials, paralleling designs already used for student-centred and school-wide programmes. Given the observational design and the single specialist setting, wider implementation would be premature. The immediate priority is replication in mainstream and other specialist schools, with larger and more diverse samples, followed by small pilot studies before any move to causal efficacy trials. Because the data come from a single London-based school, contextual and cultural influences on teacher behaviour also remain untested. Concrete next steps include longitudinal designs, sequential or lagged coding to address directionality [60], comparisons with established classroom-management frameworks, and, ultimately, multi-school intervention trials.

The findings also carry important implications for teacher training. Disruptive behaviour remains a pressing challenge in schools [5], yet many teachers report feeling underprepared [7,8]. Existing programmes are numerous but fragmented: most are grounded in behavioural psychology [58], while trauma-informed approaches [70] and social–emotional learning (SEL) programmes [71] emphasise socio-emotional development but do not directly target disruptive behaviour.

Teacher-centred MBIs may therefore function as an integrative framework, linking behavioural, trauma-informed, and SEL approaches through the shared mechanism of strengthening mentalization.

## 5. Conclusions

In conclusion, this naturalistic observational study identified associations between teacher-centred MBIs and the resolution of disruptive behaviour in classrooms; because there was no experimental manipulation, these findings indicate associations rather than evidence that the interventions were effective. Stimulating students’ mentalizing skills and reducing emotional arousal emerged as strategies associated with resolution, underscoring the importance of pacing interventions to arousal. Empathetic validation appeared to function as a supportive precursor for subsequent interventions, whereas progress-oriented validation may have reinforced behavioural de-escalation. Encouraging students to consider other perspectives of reality—through both playful and non-playful reframing—was also associated with resolution, suggesting that playfulness can act as a functional proxy for pretend play. By contrast, strategies such as joint problem-solving or task scaffolding were not associated with resolution, illustrating how easily MBT-informed practices can drift from their theoretical roots when adapted to classrooms. Although only one synergistic pairing emerged, additive effects were observed across incident severity, reinforcing the value of flexibly layering strategies.

More broadly, the findings highlight both the promise and the challenge of translating MBT principles into educational contexts. Because these associations emerged in a single specialist alternative-provision school with an embedded trauma-informed culture and near one-to-one staffing, they may not generalise to mainstream schools, to schools without trauma-informed practices, or to teachers without comparable professional preparation, and they require replication before any implications for teacher training or programme development are drawn. Effective application requires balancing theoretical fidelity with classroom feasibility. Some strategies, such as Mentalization, Down-regulation, and Playing with reality, appear well suited to this balance, while others (Validation, Collaboration, Scaffolding) hold potential but require further refinement.

## Figures and Tables

**Table 2:** Categorization of student disruptive behaviour by severity.

**Score of 1:** **Mild severity**	A score of 1 was assigned to: (a) disruptive behaviours that only briefly or minimally disrupt the classroom environment, or (b) emotional dysregulation signs that are low in emotional intensity and subtle but reflect underlying discomfort or difficulty coping.
	*Examples:* chatting to others, off-task behaviours, tapping, swinging on a chair, whistling, fiddling, making noises, not following instructions, singing, withdrawal, avoidance, and passivity.
**Score of 2:** **Moderate severity**	A score of 2 was assigned to: (a) disruptive behaviours that are more frequent or persistent and cause noticeable disruption to the classroom environment, or (b) emotional dysregulation signs that are clearly noticeable but not yet high in emotional intensity.
	*Examples:* talking over other people, wandering around, name calling or insults, taking others’ belongings, talking back disrespectfully, frustration or irritability.
**Score of 3:** **High severity**	A score of 3 was assigned to: (a) disruptive behaviours that are very frequent, persistent, or harmful to self or others, or (b) emotional dysregulation signs that are high in emotional intensity and highly noticeable.
	*Examples:* verbal or physical aggression towards self or others, throwing objects, racism, bullying, defiance, offensive language, threatening or intimidation, freezing or shutting down.

**Table 3:** Pearson chi-square (upper) and phi coefficients (lower) between interventions.

	Mentalization	Validation	Down-Regulation	Playing with Reality	Collaboration	Scaffolding
Mentalization		44.077 *** (*p* < 0.001)	41.336 *** (*p* < 0.001)	2.039 (*p* = 0.153)	14.270 *** (*p* < 0.001)	3.330 (*p* = 0.068)
Validation	0.233 *** (*p* < 0.001)		20.230 *** (*p* < 0.001)	0.022 (*p* = 0.532)	0.612 (*p* = 0.434)	0.133 (*p* = 0.715)
Down-regulation	0.225 *** (*p* < 0.001)	0.158 *** (*p* < 0.001)		2.391 (*p* = 0.122)	1.468 (*p* = 0.226)	0.173 (*p* = 0.677)
Playing with reality	0.050 (*p* = 0.153)	0.022 (*p* = 0.532)	0.054 (*p* = 0.122)		4.968 * (*p* = 0.026)	0.493 (*p* = 0.483)
Collaboration	0.132 *** (*p* < 0.001)	0.027 (*p* = 0.434)	0.042 (*p* = 0.226)	0.078 * (*p* = 0.026)		12.224 *** (*p* < 0.001)
Scaffolding	0.064 (*p* = 0.068)	0.013 (*p* = 0.715)	0.015 (*p* = 0.677)	0.025 (*p* = 0.483)	0.122 *** (*p* < 0.001)	

* *p* < 0.05. *** *p* < 0.001. Note. Degrees of freedom = 1.

**Table 4:** Frequencies and chi-square results for each intervention.

**Mentalization**						
	+		-		χ^2^ (1)	Significance
	*n*	%	*n*	%		
Resolved	118	47.6	68	12.0	124.059	<0.001 ***
Not resolved	130	52.4	499	88.0		
Total	248	100.0	567	100.0		
**Validation**						
	+		-		χ^2^ (1)	Significance
	*n*	%	*n*	%		
Resolved	68	44.2	118	17.9	49.064	<0.001 ***
Not resolved	86	55.8	543	82.1		
Total	154	100.0	661	100.0		
**Down-regulation**						
	+		-		χ^2^ (1)	Significance
	*n*	%	*n*	%		
Resolved	90	41.7	96	16.0	59.253	<0.001 ***
Not resolved	126	58.3	503	84.0		
Total	216	100.0	599	100.0		
**Playing with reality**						
	+		-		χ^2^ (1)	Significance
	*n*	%	*n*	%		
Resolved	55	39.0	131	19.4	25.357	<0.001 ***
Not resolved	86	61.0	543	80.6		
Total	141	100.0	674	100.0		
**Collaboration**						
	+		-		χ^2^ (1)	Significance
	*n*	%	*n*	%		
Resolved	22	38.6	164	21.6	8.658	0.003 *
Not resolved	35	61.4	594	78.4		
Total	57	100.0	758	100.0		
**Scaffolding**						
	+		-		χ^2^ (1)	Significance
	*n*	%	*n*	%		
Resolved	7	38.9	179	22.5	2.698	0.100
Not Resolved	11	61.1	618	77.5		
Total	18	100.0	797	100.0		

* *p* < 0.05. *** *p* < 0.001.

**Table 5:** Cross-classified multilevel logistic models of the use and combination of interventions predicting incident resolution.

	Model 1				Model 2			
Fixed Effects	*OR*	95% CI		*p*	*OR*	95% CI		*p*
		*LL*	*UL*			*LL*	*UL*	
Intercept	0.112	0.057	0.217	<0.001 ***	0.137	0.067	0.282	<0.001 ***
Intervention (ref = 0)								
Mentalization	5.183	3.448	7.791	<0.001 ***	3.201	1.659	6.177	<0.001 ***
Validation	2.368	1.507	3.723	<0.001 ***	1.735	0.705	4.266	0.230
Down-regulation	3.753	2.428	5.801	<0.001 ***	3.603	1.787	7.264	<0.001 ***
Playing with reality	3.411	2.088	5.574	<0.001 ***	2.665	1.250	5.682	0.011 *
Collaboration	1.544	0.790	3.018	0.203	1.290	0.265	6.265	0.752
Intervention × Intervention								
Mentalization × Validation					2.295	0.871	6.045	0.093
Mentalization × Down-regulation					0.953	0.398	2.283	0.914
Mentalization × Playing with reality					3.060	1.042	8.988	0.042 *
Mentalization × Collaboration					2.802	0.562	13.965	0.208
Validation × Down-regulation					1.130	0.422	3.023	0.807
Validation × Playing with reality					0.699	0.181	2.705	0.603
Validation × Collaboration					0.287	0.053	1.557	0.148
Down-regulation × Playing with reality					1.158	0.360	3.728	0.815
Down-regulation × Collaboration					1.940	0.402	9.373	0.409
Collaboration × Playing with reality					0.179	0.031	1.024	0.053
Random effects	Variance				Variance			
	Estimate		*p*		Estimate		*p*	
Teacher identifier	0.059		0.517		0.084		0.453	
Student identifier	0.326		0.124		0.326		0.113	
Incident number	5.47 × 10^−6^		0.622		6.29 × 10^−6^		0.615	

* *p* < 0.05. *** *p* < 0.001. Note. OR = odds ratio. ref = reference group. CI = confidence interval; LL = lower limit; UL = upper limit. For each intervention, the reference category (0) denotes incident parts in which that specific intervention was not used; each odds ratio therefore contrasts parts where the intervention was present with otherwise comparable parts where it was absent, rather than contrasting any intervention with none.

**Table 6:** Cross-classified multilevel logistic model of the number of interventions predicting incident resolution.

Fixed Effects	*OR*	95% CI		*p*
		*LL*	*UL*	
Intercept	0.111	0.058	0.212	<0.001 ***
Number of interventions	3.293	2.690	4.029	<0.001 ***
Severity (ref = Mild severity)				
Moderate severity	0.859	0.531	1.390	0.651
High severity	0.584	0.344	0.992	0.047 *
Number of Interventions × Severity				
Number of interventions × Moderate severity	1.075	0.655	1.765	0.775
Number of interventions × High severity	1.440	0.880	2.357	0.147
Random effects	Variance			
	Estimate	*p*		
Teacher identifier	0.073	0.466		
Student identifier	0.316	0.121		
Incident number	3.37 × 10^−6^	0.671		

* *p* < 0.05. *** *p* < 0.001. Note. OR = odds ratio. CI = confidence interval; LL = lower limit; UL = upper limit.

## Data Availability

The data are available on request from the corresponding author due to ethical restrictions on sensitive clinical data from children.

## Supplementary Materials

The following supporting information can be downloaded at: https://www.mdpi.com/article/10.3390/children13070911/s1, S1: The SPSS syntax for the cross-classified multilevel models; S2: The full coding manual and operational definitions for the six MBIs and the severity framework; S3: Worked examples of recorded observation notes coded under each intervention category.
