# Supplementary material for: When Teachers Mentalize: A Mixed-Methods Analysis of Teacher Responses to Disruptive Classroom Behaviour
Source: Children (Basel). 2026 Jul 9;13(7):911. doi: 10.3390/children13070911 (PMC13406815; doi:10.3390/children13070911)
Supplement: Supplementary file 1 [file children-13-00911-s001.zip › children-4382286-supplementary.pdf]

Supplementary Materials

When Teachers Mentalize: A Mixed-Methods Analysis of Teacher Responses to Disruptive Classroom Behaviour

S1. SPSS Syntax for the Cross-Classified Multilevel Models

The cross-classified generalized linear mixed models reported in the manuscript were estimated in IBM SPSS Statistics (Version 30) using the GENLINMIXED procedure, with a binomial distribution and logit link. Incident resolution (0 = unresolved, 1 = resolved) was the dependent variable, and teacher, student, and incident identifiers were entered as crossed random effects.

```
* Generalized Linear Mixed Models: interventions predicting incident resolution.
GENLINMIXED
  /FIELDS TARGET=Resolved0lo1ken TRIALS=NONE OFFSET=NONE
  /TARGET_OPTIONS DISTRIBUTION=BINOMIAL LINK=LOGIT
  /FIXED EFFECTS=ment vali dr play colla sca OverallSeverity123 USE_INTERCEPT=TRUE
  /RANDOM EFFECTS=TeacherN StudentN IncidentNumber USE_INTERCEPT=TRUE
    COVARIANCE_TYPE=VARIANCE_COMPONENTS SOLUTION=FALSE
  /BUILD_OPTIONS TARGET_CATEGORY_ORDER=ASCENDING INPUTS_CATEGORY_ORDER=ASCENDING
    HCONVERGE=0.00000001(RELATIVE) MAX_ITERATIONS=100 CONFIDENCE_LEVEL=95
    DF_METHOD=RESIDUAL COVB=MODEL SCORING=0 SINGULAR=0.000000000001
  /EMMEANS_OPTIONS SCALE=ORIGINAL PADJUST=SEQBONFERRONI.
```

S2. Operational Definitions of the Coding Scheme

Table S2.1. The six mentalization-based interventions (MBIs) and their operational definitions, applied to each teacher response.

Intervention	Operational definition
**Mentalization**	Mentalization is central to a mentalization-based approach. It involves understanding and interpreting the student's mental states (e.g., thoughts, emotions, desires), rather than focusing solely on their behaviour. It also involves the teacher sharing their own mental states to model self-awareness and guide their interactions with the student.
**Validation**	Validation involves accepting and acknowledging the student's subjective emotional experience, regardless of its divergence from objective reality. It communicates to the student that their feelings and perceptions are understandable and legitimate. This intervention promotes a positive, hopeful, "can-do" attitude.
**Down-regulation**	Down-regulation involves the teacher's efforts to reduce the student's emotional arousal to a more manageable and tolerable

	level before engaging in mentalization. This may include soothing language, a soft tone of voice, non-threatening body language, or other calming techniques. This intervention fosters a sense of safety and security within the student.
**Playing with reality**	Based on the Playing with reality series [43–46], this intervention involves helping the student shift from a rigid, concrete perception of reality (psychic equivalence) to a more flexible one. The teacher may use playfulness or gentle humour to encourage exploration of alternative perspectives. This creates a safe environment for the student to consider that reality can be experienced and viewed in different ways.
**Collaboration**	Collaboration refers to the teacher involving the student in the co-construction of meaning and action. By positioning the student as an active partner in problem-solving or joint activity, this intervention fosters a sense of alliance between the teacher and the student and enhances the student's sense of agency.
**Scaffolding**	Based on Vygotsky's zone of proximal development [47], scaffolding involves tailoring strategies to the student's current stage of cognitive and emotional development. The teacher provides the support necessary to enable the student to perform a task just beyond their current abilities, gradually reducing support as the student gains mastery.

Table S2.2. Severity levels of disruptive behaviour.

**Level**	**Definition and examples**
**Score of 1: Mild severity**	A score of 1 was assigned to: (a) disruptive behaviours that only briefly or minimally disrupt the classroom environment, or (b) emotional dysregulation signs that are low in emotional intensity and subtle but reflect underlying discomfort or difficulty coping. Examples: chatting to others, off-task behaviours, tapping, swinging on chair, whistling, fiddling, making noises, not following instructions, singing, withdrawal, avoidance, passivity.

**Score of 2: Moderate severity**	A score of 2 was assigned to: (a) disruptive behaviours that are more frequent or persistent and cause noticeable disruption to the classroom environment, or (b) emotional dysregulation signs that are clearly noticeable but not yet high in emotional intensity. Examples: talking over other people, wandering around, name calling or insults, taking others' belongings, talking back disrespectfully, frustration or irritability.
**Score of 3: High severity**	A score of 3 was assigned to: (a) disruptive behaviours that are very frequent, persistent, or harmful to self or others, or (b) emotional dysregulation signs that are high in emotional intensity and highly noticeable. Examples: verbal or physical aggression towards self or others, throwing objects, racism, bullying, defiance, offensive language, threatening or intimidation, freezing or shutting down.

S3. Worked Examples of Coded Observation Notes

The following anonymised excerpts, drawn from the recorded classroom observations, illustrate one example of each intervention as coded. Speaker roles are given in brackets; no students, staff, or classes are identified.

Mentalization. The teacher shares their own mental state, modelling reflective thinking about the situation. *"Can you please pull your chair up? I'm scared you'll hurt yourself." [Teaching Assistant, Female]*

Validation. The teacher empathically acknowledges the legitimacy of the student's subjective experience. *"I'm sorry, that's not very kind, is it? He should've asked." [Teacher, Male]*

Down-regulation. The teacher lowers the student's arousal through soothing language and the offer of a calmer space. *"Do you want to go somewhere where it is nice and quiet?" [Teacher]*

Playing with reality. The teacher uses gentle humour to reframe a tense moment and invite a more flexible perspective. *"Unfortunately, a table is always going to win, so don't square up." [Teacher, Female]*

Collaboration. The teacher invites the student into joint planning, using "let's" language to convey partnership. *"Let's make a plan for the day together!" [Teacher, Male]*

Scaffolding. The teacher breaks the task down by first clarifying the source of the difficulty. *"What don't you understand?" [Teacher]*
